# Moral Distress Consultation Services: Insights from Consultants

**DOI:** 10.1007/s10730-024-09535-4

**Published:** 2024-08-03

**Authors:** Vanessa Amos, Phyllis Whitehead, Beth Epstein

**Affiliations:** 1 School of Nursing, University of Virginia, Charlottesville, VA USA; 2 Carilion Roanoke Memorial Hospital, Roanoke, VA USA

**Keywords:** Moral Distress, Organizational Ethics, Ethics, Nursing, Leadership

## Abstract

Moral distress reflects often recurrent problems within a healthcare environment that impact the quality and safety of patient care. Examples include inadequate staffing, lack of necessary resources, and poor interprofessional teamwork. Recognizing and acting on these issues demonstrates a collaborative and organizational commitment to improve. Moral distress consultation is a health system-wide intervention gaining momentum in the United States. Moral distress consultants assist healthcare providers in identifying and strategizing possible solutions to the patient, team, and systemic barriers behind moral distress. Moral distress consultants offer unique perspectives on the goals, successes, areas for improvement, and sustainability of moral distress consultation. Their ideas can help shape this intervention’s continued growth and improvement. This qualitative descriptive study features 10 semi-structured interviews with moral distress consultants at two institutions with longstanding, active moral distress consultation services. Themes from consultant transcripts included consultant training, understanding the purpose of moral distress consultation, interfacing with leadership teams, defining success, and improving visibility and sustainability of the service. These findings describe the beginnings of a framework that organizations can use to either start or strengthen moral distress consultation services, as well as the first steps in developing an evaluation tool to monitor their utility and quality.

**Supplementary Information:**

The online version contains supplementary material available at 10.1007/s10730-024-09535-4.

## Introduction

Moral distress (MoD) occurs when a healthcare provider (HCP) recognizes what they believe is an ethically appropriate action or professionally appropriate obligation (e.g., minimizing unnecessary suffering or providing high-quality care) but is constrained from taking it due to some external force (Hamric et al., 2015; Jameton, 1984; Varcoe et al., 2012). It is often reflective of problems within a healthcare work environment, such as inadequate staffing, lack of resources, and/or poor interprofessional teamwork (Epstein et al., 2019; Karanikola et al., 2014). Because MoD occurs across professions and healthcare settings, an organizational commitment to recognizing and addressing its causes is critical. This is especially true in the wake of the COVID-19 pandemic, where rethinking and redesigning healthcare delivery as well as assessing the organizational environment in which healthcare is delivered is deeply needed. Moral distress consultation (MDC) is emblematic of such an approach, and is among the few MoD interventions that have shown specific promise in aiding those who experience MoD (Amos & Epstein, 2022; Dacar et al., 2019; Epstein et al., 2021).

Borne out of clinical ethics consultation (CEC), MDC has been implemented in a subset of hospitals since 2006 (Hamric & Epstein, 2017). Both CEC and MDC can be utilized for patient related concerns, but, in contrast to CEC, the primary goal of MDC is to assist HCPs in identifying those patient-, team-, and system-level barriers keeping them from achieving their professional obligations (Hamric & Epstein, 2017). Key to the MDC process is also strategizing possible solutions to these barriers (Hamric & Epstein, 2017). MDC consultants offer unique perspectives on the realities and potential of MDC, but little is known about their lived experiences and perspectives. Their views are valuable, especially when trying to understand the sustainability of MDC within healthcare systems. Given the high-stakes clinical situations in which MDC tend to be involved, evaluation of these services must also be considered. Various CEC evaluation tools are available, including those that obtain feedback from users (such as HCPs, patients, families) or those that evaluate the CEC process and outcomes (Au et al., 2018; Bell et al., 2022; *Core Competencies for Health Care Ethics Consultation*, 2010; Fox et al., 2006; Hern, 1990; Orr et al., 1996; Pearlman et al., 2016). MDC evaluation tools do not currently exist. This paper is part of a larger, multi-method study to better understand MDC from the perspectives of various stakeholders and describes MDC consultants’ perspectives that may help organizations develop MDC services and includes suggestions for a future evaluation tool.

## Methods

### Setting

This study took place at two mid-Atlantic Level 1 academic regional referral centers in the southeast United States from February through June 2023. Both facilities support greater than 650 inpatient beds, across over 30 adult and pediatric critical and acute care units. Both organizations also have well-established MDC services (founded in 2006 and 2015) with trained consultants who follow defined processes (outlined below). Consultant membership at these two institutions’ MDC services does fluctuate, but in June of 2023, there were eight active MDC consultants at one institution and six at the other. Also, as of June 2023, 170 consults had been completed at one facility, 86 at the second. IRB approval was obtained at both institutions.

### Moral Distress Consultation

At both institutions, MDC is a health system-wide service accessible through the institution’s CEC services using a specific process to assist HCPs in identifying and mitigating morally distressing situations (Hamric & Epstein, 2017).

#### Process

MDC can be requested through a 24/7 pager service by any HCP. One-hour consults are then scheduled and coordinated to maximize multidisciplinary attendance. The consults are conducted in person, via a secure online meeting platform, or both. Two trained consultants (one facilitator, one scribe) follow a structured process, including defining MoD, identifying the underlying causes of MoD, determining barriers to action, and brainstorming with participants possible strategies to overcome the identified barriers (Hamric & Epstein, 2017). The scribe documents the major points of the discussion, but no protected health information is accessed, nor is any documentation pertaining to a MDC placed in a patient’s chart (Hamric & Epstein, 2017). Summaries are provided to the consult requestor to promote implementation of the strategies identified during the consult. Whether particular strategies are implemented is not currently being tracked, though consultants are available for follow-up if requested. Review of MDCs occurs during weekly or bi-monthly CEC meetings attended by all MDC and CEC consultants.

#### Training

Training in MDC may vary across institutions, but for the two MDC services described in this paper, experienced clinical ethics consultants interested in MDC are first provided readings on MoD and are encouraged to attend a 4-day training session on CEC and MDC. This intensive training is offered annually at one of the two organizations in this study and includes a full day dedicated to MDC, in addition to debriefing past consults from CEC and MDC services. Trainees then enter a period of observation of scheduled MDCs and participate in post-consult debriefings. New consultants can also serve as a scribe for scheduled consults. The length of the training period depends on trainee confidence and on the frequency of MDC requests (e.g., few MDC requests could equate to a given trainee taking “longer” to move to facilitator role). Readiness occurs when the trainee expresses familiarity and confidence about MDC and the facilitator (a senior consultant) believes the trainee has demonstrated the knowledge and ability to act as a facilitator. When ready, new consultants lead consults, first with a senior consultant serving as a scribe for support and backup, and then partnered as MDC staffing allows. Although not optimal, it is possible for a single consultant to both facilitate and scribe for a given consult.

### Participants

MDC consultants were invited to participate over email with the following inclusion criteria: being a current or recent past MDC member at one of the identified institutions (e.g., having been a consultant within the past five years). Exclusion criteria included being the director of the consult service. All consultant participants provided written consent for a recorded 30-45-minute, virtual, semi-structured interview.

### Data Collection

The semi-structured interview guide (Supplemental Table 1) was designed to gain insight on an overarching question: “What is your experience of the moral distress consultation service, inclusive of how it functions and how it is sustained as an intervention?” All consultants gave permission for subsequent use of deidentified interview data. The data were stored securely online, and access was limited to research team members. In order to protect privacy and avoid identifiability, the consultants’ specific educational backgrounds were not collected, and all personal or institutional identifiers were removed during the transcription process. Data collection continued until data saturation (Guest et al., 2006; Hennink & Kaiser, 2022) was reached. All interviews and transcriptions were completed by the primary author. Transcription took place within 48 h of each interview and collected field notes were used to provide further rich detail (Davies & Dodd, 2002).

### Analysis

**Fig. 1:**
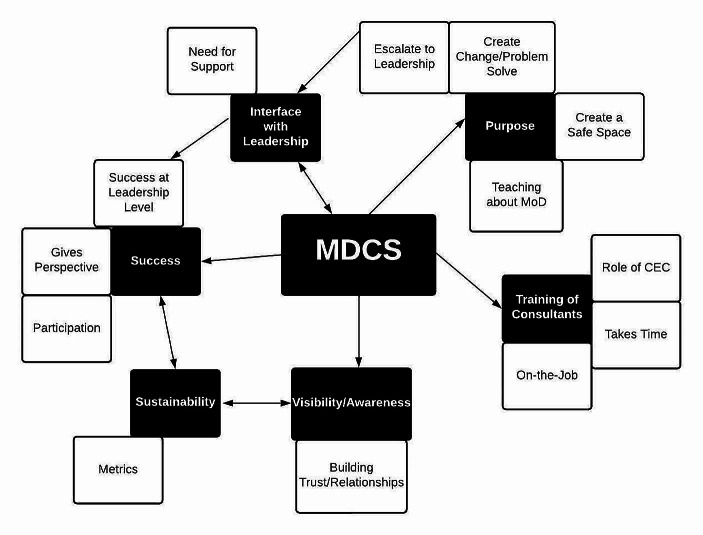
Identified themes and categories *Note*: While most themes were described as having unidirectional connections, several also suggested interdependency. A bidirectional arrow was used to reflect this difference

Deidentified transcripts were transferred and analyzed first using inductive coding (Saldana, 2021) at the semantic level (Braun & Clarke, 2006). This was completed by the primary author using Lumivero’s (2023) Nvivo software (Version 14). Beginning at the semantic level allows for the consultants’ words to dictate the molding of the data, rather than the primary author’s (Saldana, 2021). The data were then reanalyzed using a deductive constructionist view (Braun & Clarke, 2006) to take into account the primary author and their sociocultural experience of MoD in the hospital setting. The primary author is a healthcare provider, but not a MDC consultant. This secondary analysis also used a targeted, a priori code list rooted in the semi-structured interview guide (Supplemental Table 1). This dual, layered approach enhanced the rigor of the data through the continued recursive process found in quality thematic analyses (Braun & Clarke, 2006). Finally, while the primary author developed a coding frame consisting of particular codes in alignment with their analysis (O’Connor & Joffe, 2020), all deidentified transcripts were also independently coded by another researcher of similar standing and experience using open coding. The two researchers thus created separate coding frames which were then compared and discussed until an intercoder agreement (Campbell et al., 2013) was reached. Possible themes were then discussed and ultimately translated to a thematic map (Braun & Clarke, 2006; see also Fig. 1). This thematic map was then shared, discussed, and iterated with the remaining research team members.

## Results

### Demographics

Of the 12 email invitations sent to current and former MDC service consultants, 10 elected to participate, an 83% response rate. Seven consultants were from one institution, three from the second (Table 1). Data saturation (Guest et al., 2006; Hennink & Kaiser, 2022) was believed to have been reached by interview eight, though additional interviews were completed because of willing participants.

Participants reported an average of 22 years of healthcare experience (range: 10 years to 40+; see also Table 1). In addition, seven of the 10 consultants had active, direct patient care duties in addition to their MDC role. Finally, all consultants stated they had been or are currently active in their hospital’s CEC service.

**Table 1:** Demographics

Trait	Consultant Count
Age	
35–45	4
46–55+	6
Highest Degree Obtained	
Masters	2
Doctorate (PhD, DNP, MD, etc.)	8
HCP Type	
Nursing Field (RN, APRN)	4
Other (Physicians, Social Workers, etc.)	6
Tenure HCP	
10–19 years	4
20–25 years	3
26–30 + years	3
Tenure MDC	
0–4 years	4
5–10 years	4
11–15 + years	2

### Identified Themes

Identified themes included: training and introduction to MDC, defining MDC purpose, interfacing with leadership, defining MDC success, and visibility and sustainability (Fig. 1; see also Supplemental Table 2).

#### Training and Introduction to the MDCS

Participants described several modalities of training to become consultants. All (*n* = 10) explained their work in MDC began with experiences and education fostered by their home institution’s CEC service. Most then described “on-the-job” training. For example, from Consultant 3494:

With clinical ethics, that can also be on the job as well for many folks. But I was trained in it and also have a degree. So, I was always taught how to do clinical ethics. Where moral distress is more on the job training.

While this informal learning was more commonly reported than more specific MDC education (e.g., education received from an ethics or MoD conference, reading published MoD research studies, or ethics-focused undergraduate or graduate courses), several consultants did explain how more formal training was valuable. For example, Consultant 3636:

I’ve done additional ethics training, meaning the intensive that we offer, which has some moral distress in it, and the semester long class, which was the graduate level healthcare ethics consultation class, which I think had moral distress in it. And then, you know, other random, assorted courses. The HEC-C training has a little bit of it, but not very much from my recollection.

Additionally, observation was noted to be useful for trainees, as well as acting as a scribe with an experienced consultant serving as the facilitator. Several consultants commented they required years as active MDC members before feeling comfortable as a facilitator, like Consultant 1017:

I went to several consults. I can’t necessarily put a number on it, but it was probably at least ten. And [how it] started out, I just watched a few, and then I scribed a few. And really just in the last, like two or three years, I’ve actually felt somewhat comfortable facilitating them on my own.

#### Defining Purpose

When queried about the purpose of MDC, consultants highlighted three key points: to create a safe space, to provide MoD education, and to collaborate with participants to develop plans to alleviate MoD. Consultants described how providing a safe space during MDC allowed participants to feel heard and supported. Consultant 2556 said “[…] we give them a safe space to talk to, we give them a space to participate in brainstorming. Allowing people to feel engaged in that way, I think, is helpful because you start to feel powerless.”

Teaching about MoD included consultants defining MoD, explaining why unresolved MoD is concerning and how MDC represents an organizational commitment to creating a moral community:

I would say that it [the MDC service] is a mechanism […] for mitigating moral distress both in the sense of addressing current problems and issues, which are often systemic, [but also] for educating staff along the way and empowering them, and as a mechanism for making the institution a more cohesive moral community. (Consultant 1324)

Developing plans to alleviate MoD was mentioned by all consultants (*n* = 10). One example of problem-solving at the unit level included brainstorming with staff about how they could create change in existing practices, such as through interprofessional rounds or scheduled debriefings after significant patient events. These strategized solutions could also focus on larger scale changes, including escalation to upper-level leadership, seeking changes in established hospital policy or even organizational culture shifts around MDC requests:

I think staff have seen us enact change. Especially from the policy standpoint. I also think there have been slow wins as far as culture. We’re also seeing consults from units, like this year, that the last couple of years we would have never been consulted on before. (Consultant 1017)

#### Interface with Leadership

Helping leaders understand what MDC is, how the service can benefit from their support, and how building relationships with consultants can be meaningful at the organizational level was mentioned by eight consultants. Consultant 1778 explained:

So, it’s an interesting thing, because the phenomenon [of MoD] in general, if you just raise it conceptually, I think most institutions don’t want any part of it. Then when you actually have something that’s in practice [like MDC], and someone says, “here’s how it was used,” or “this is how it was beneficial,” they think “oh, well, okay, I can see that.” Like, “that’s a good thing. We don’t want to get rid of that.”

Consultants also noted that how having trusting relationships with leaders was crucial in ensuring escalation from a consult was possible and fruitful:

And I think the important thing is being able to say to them [the upper leadership team], “This is not okay.” And staff, or even unit managers […] they’re not always positioned to be able to be that forthright, and say, “You [upper-level leadership member], I’m talking to you. Not okay. This is on your watch, and you’re responsible. You’re a member of a moral community.” (Consultant 1324).

Consultant 2080 explained upper-level leaders are not opposed to MDC or its function within a health system: “I’ve never gotten the impression that [MDC director] is fighting for the existence of the moral distress consult service [with upper-level leadership teams].” Consultant 3494 echoed this feeling by saying, “[upper-level leaders] find it [MDC] very valuable and I don’t think I would have any pushback from leadership saying that it’s not.”

Yet, several consultants reflected on hopes for more leadership support:

And I wish that they [leadership team members] would see moral distress, not as a negative, I mean moral distress is never positive, but I wish that they would see that instead of the staff, you know, participating in these consults, instead of seeing them as whiners or complainers, or whatever, I wish they would see these people are putting their necks on the line to speak out loud about injustice that they see in the system. (Consultant 1017)

They followed this with:

I think I would want to say to our leadership that I really hope that they will take it upon themselves to support us more. Because really what we want to be is their eyes and ears to enact positive change across the organization. (Consultant 1017)

How this leadership support could be demonstrated was variable, and sometimes not specific, but Consultant 3636 said, “I think having that formal recognition from leadership and that allotted time [protected and compensated time dedicated to MDC] is important. I do think that’s incredibly important.” Consultant 1324 further described one way this had been achieved:

So, we [hospital leadership and MDC members] looked at a couple of models, and we, you know we drilled it all down to here’s the FTEs that we need, and you know, here are the people, and we’re going to do it at this percent effort. And so that took a fair amount of work, but it’s done, you know. And it’s not rocket science. It’s just sitting down and doing.

### Defining Success

Participating consultants saw MDC as successful when a consult personally affected MDC participants, created change within the organization, and/or promoted MDC sustainability. Consultants noted that a “shift in perspective” occurred when MDC participants recognized their concerns were being heard and that MDC helped to identify those concerns:

[…] you can just tell the difference in people and their voices and how they perceive. And I think just giving people that voice and realizing the emotional and mental burden sometimes you’re relieving from people because they really care about their patients. And just letting them know that we’re really trying to take that next step and you’re being heard. (Consultant 3283)

Another example comes from Consultant 1558, who said, “I think part of it is just a space to give people like I said, a voice. [For those] who didn’t feel like they had one. And sometimes that does a lot of good right then.” This change in perspective proved integral not only to the perceived well-being of the staff, but also in terms of gathering learning techniques to apply to future morally distressing situations. Consultant 1558 explained how the consult service may not serve as the pivotal change agent but could be the conduit through which change can occur:

Because the parties involved in the moral distress consult may not only be able to fix some of the systemic stuff, but then it would be perpetuated, right? And you use some of the tools from that so that you don’t have to call again. You’re just like, “Okay, wait a second, let’s just think about this. Remember what we did before.”

Regarding success at the organizational level, consultants described a difficulty in establishing clear quantitative metrics:

Unfortunately, a lot of things are subjective. I feel so much of what happens and what is done, we can’t show. Like [in other role] I can say, “I saved your budget this many dollars by changing to this.” We can’t do that with this. The intangibles are tremendous. (Consultant 3283)

Consultant 1017 elaborated on this idea with their description of how they hoped upper-level leadership would view MDC:

I would want them to look at how we have improved staff safety, how we have improved communication among teams, how we have improved the language that attendings and residents use when they’re talking to patients and their families about goals of care discussions. I would want them to look at a unit where nurses feel that they can walk up to an attending and say, “I don’t think this care plan is right. And here’s why.” Where we can have an open dialogue with each other as a community.

Utilization of MDC did appear among several transcripts (*n* = 3) as one source of quantitative measurement and an indicator of sustainability. For example, Consultant 1032 stated:

I think the other thing the system would need to see is a utilization of the moral distress [consultation service], right? You know, if they saw it being utilized more, they may be like, “Wow, this is a service that must be needed because our people are using it.” So, I think they would need to see that.

#### Visibility and Sustainability

In response to interview questions about MDC visibility and sustainability, several consultant participants noted the importance of relationships between leaders and MDC consultants to foster consistent exposure of MDC to hospital staff. For example, Consultant 1558 said, simply, “To be sustainable, it’s going to have to be just the awareness of the service.” Visibility often hinged on having participated in a consult or knowing a particular consultant. For example, Consultant 3494 described how integral certain consultants were:

Relationships go a long way. And I know [MDC member] has a good relationship with a lot of clinicians, so they feel very free to reach out to them. I think that’s a big part. Because of their presence and their knowledge, folks reach out to them.

Staff turnover, however, was mentioned as a barrier to visibility and sustainability, suggesting future MDC advertisement and upper-level leadership support would need to be consistent to ensure MDC success. Consultant 1778 explained:

With an academic health institution, you have a tremendous amount of turnover. And of course, we’ve seen that turnover exponentially rise since the COVID years. You can go in one month and do a presentation on the moral distress consult service and the ethics consult service. You can do the whole song and dance. You can show up to meetings, everything. And six months later, you’ve got an entirely new group of people and they don’t know. So, you either have to do it every six months, or rely on a few key people in those units to still say, “Hey, you know, we have one of these things.”

And Consultant 1032 expanded on the need for upper leadership support:

Maybe there needs to be more people involved then, you know, from the higher ups because I do feel like [MDC member] takes a lot of it and [they do] a great job, but [they] will retire one day or get burned out. And [they need] help with that.

The requirements placed on MDC service members were then further elaborated on by some, and often described as needing of support, both internally and externally, as well as a continued demand for more formalized training. For example, Consultant 1324 explained:

And you can get the science and doing it right. But the art of doing it. And so that’s part of training. And so, I think the more that we can formalize training and expectations and metrics the better we’ll do. And moving forward across the country in terms of implementing these sorts of things.

When pressed about more specific needs for sustainability, Consultant 2556 highlighted the need for internal debriefings:

I think, longevity wise, to keep us going. I think we [as consultants] need a space [to debrief]. However we do that, whether it’s once a month we have a different meeting or some time is set aside in the ethics consult services meetings, we need to set aside some time to actually process these [MDCs]. We’re hearing a lot of stuff. And I feel like we probably need to debrief that.

The importance of physician involvement in MDC services was also mentioned when asked about sustainability and visibility. For example, Consultant 1778 stated, “I think as long as we’re going to have physicians and nurses together, you’ve got to have both of them at the table for this, or else it’s going to continue to limp along.” Joining the MDC team was not described as difficult nor were any specific barriers highlighted. For example, Consultant 3283 described how they reached out:

I just picked up the phone and called [MDC member] and was like, “Hey, I have an interest in this.” And they said, “That’s wonderful. Let’s work together. Let’s do this.” That doesn’t happen a lot. Just people that want to grow the service and want to take care of it.

## Discussion

MDC is an effective intervention to address MoD and strategize solutions around its causes (Epstein et al., 2021). This qualitative descriptive study describes the experiences of MDC consultants, which adds to the understanding of this intervention’s functionality. The themes identified in this study also provide guidance to those considering developing such a service and suggest key components for evaluation.

### Consultant Education and Training

Contrary to what is currently perceived, consultants are not necessarily nurses. In fact, participants in this study were varied in their professional backgrounds and over half (*n* = 6) were not nurses. Eight held doctoral degrees and all were experienced healthcare providers (with 10 years or more in healthcare). While these specific education and experience backgrounds are not necessary requirements for MDC, they are important factors to consider for those organizations looking to develop MDC services. MoD is well known to all HCPs and the composition of the consultant team should reflect that. Similar advantages of professional diversity have been reported in CEC (Ulrich & Grady, 2023). At both institutions, too, experience in CEC was required. This study’s MDC services existed within CEC services, which fosters communication and collaboration among both teams and ensures that MDC consultants are grounded in CEC core competencies (*Core Competencies for Health Care Ethics Consultation*, 2010). Finally, this study’s consultant participants did not all have protected time for their participation in MDC. This is similar to CEC and other programs within hospital systems, where time may be protected or can be volunteered. Because MDC does require significant time and dedication, this commitment balance must be carefully considered, especially if MDC members have many obligations and/or requirements outside of MDC.

When considering consultant education and training, this study’s participants noted it can involve multiple components. Understanding concepts such as MoD, moral residue, and moral community can build on a consultant’s CEC knowledge (e.g., end of life issues, privacy & confidentiality, etc.) and exposure to the MDC process through observation, debriefs, and taking on consultant roles (as scribe or facilitator) builds confidence and competence. Effective CEC services echo the need for formalized training (Fox et al., 2022; Fox, Tarzian, Fox et al., 2022a, b) and several CEC evaluation tools address this specifically (Crico et al., 2021; Pearlman et al., 2016). Therefore, formalized training, either through internal hospital based or external educational sources, is critical to ensure MDC consistency and quality.

### MDC Purpose and Process

Consultant participants identified a central purpose of MDC: to guide MDC participants in strategizing actionable, system-centered solutions. While documentation and follow-up of these strategized solutions would be of benefit, this currently lacks a formal procedure. This is an avenue of future study and will give more shape to potential evaluation tool development. For this study’s consultants, the goals of MDC could also be multi-faceted and extend beyond MDC’s stated purpose. For example, consultants identified providing a safe space for its participants and MoD education as important. This could be because many HCPs do not recognize MoD as real and instead blame themselves for not being able to handle healthcare workplace stressors. Therefore, at every MDC, not only are efforts made to provide a space for open and honest communication but also education about MoD, including differentiating MDC from other services such as CEC or employee assistance programs. Consultants at this study’s institutions also teach beyond MDCs, providing instruction to nurse residents, physician residents, leadership teams, unit staff, and medicine and nursing students. Effective education is echoed in CEC, especially when preventing role confusion and other barriers to accessing CEC services (Fox et al., 2022).

### MDC Success Outcomes

As with CEC, where engagement across staff levels and leadership teams gives rise to a sustainable and successful services (Cunningham et al., 2019; Fox et al., 2022a; Fox, Tarzian, Fox et al., 2022a, b), this study’s consultants identified collaborating with supportive leaders and ensuring a well-used service can lead to MDC success. Thus, quantitative measures such as number of consults per year or quarter and/or their associated MoD cause(s) as well as measuring engagement with leadership teams (through their presence at MDCs or meetings generated with them because of MDC) are important evaluation elements to consider. Previous studies have indicated the MDC is a useful mechanism for staff experiencing MoD (Hamric & Epstein, 2017, Epstein et al., 2021) and this provides further impetus for ensuring leaders understand the role of MDC and that their active support is an overall benefit to the organization’s construction of a moral community. Beyond straightforward measures of MDC counts, consultants expressed the challenge of finding a metric, either to gauge how to improve MDC and/or to justify its continuation to leadership teams. Quality assessment is subjective, so attributing numerical scales or other quantitative measures is often difficult (Cunningham et al., 2019; Donabedian, 1966). Further, MDC is strategy driven, with organizational culture and team relationships often playing fundamental roles in crafting potential solutions. This dynamic adds to the difficulty in generating quantitative data. To this point, developing a quality improvement collaborative or consortium to establish agreed-upon markers of success may prove useful (Cunningham et al., 2019; Schouten et al., 2008). This approach would not be without its own problems, as data from quality improvement collaboratives can be limited by their size, generalizability, and sustainability (Mittman, 2004; Solberg, 2005). However, the relatively small population of active MDC services lends itself to a more manageable and collaborative space and the potential for tracking actionable strategies implemented post-MDC. In whatever capacity a new outcome measure may be developed, traditional quantitative metrics may not be fully helpful in capturing the entire breadth of MDC.

## Conclusion

This study provides useful insights for organizations interested in developing or sustaining MDC. MDC provides HCPs, leaders, and other stakeholders opportunities to collaborate as a team and be creative in identifying ways to solve patient-, team-, and systems-level problems that tend to recur. When integrated into an organization, MDC becomes a contributor to the organization’s moral community (Liaschenko & Peter, 2016) by sustaining a mechanism to work through difficult issues and supporting staff who see problems impacting patient care quality and want to be part of the solution. Hamric and Epstein (2017) noted that, “the goal of the MDCS is not to eradicate moral distress, but to address it when it occurs and intervene early so that those providing care are empowered to act and know there are resources to help them in difficult situations…” (p. 141). Focusing on MDC as a contributor to a moral community is the place to start when designing or strengthening MDC at any organization.

### Limitations

The results described here represent a small sample of healthcare providers who also serve (or have served) as MDC consultants. We acknowledge the potential for self-selection bias among these motivated and engaged consultants. However, given the small fraction of each institution’s staff who serve on the MDC service as well as the 83% response rate from those invited, we feel the data are representative of these MDC teams’ perspectives. We also recognize the ideas presented here are not generalizable, though this study was conducted across two institutions with active, longstanding services.

### Implications for Future Work

Unlike CEC, there are currently no formalized core competencies for MDC. Future work should include collaboration with and among other MDC services to evaluate content knowledge, processes, and practices that are critical for effective consultation. Core competencies can then be developed and implemented, and once established, perhaps added to the Joint Commission’s mandate that hospitals for formal mechanisms to address clinical ethical dilemmas (*Joint Commission International Accreditation Standards for Hospitals Including Standards for Academic Medical Center Hospitals*, 2022). The themes from this study (Fig. 1) could be a springboard for such work. Developing and testing an evaluation tool is another important step. While likely a challenge, the work is still worth doing. To be successful, MDC evaluation should encompass the complete MDC process, including its usefulness to those who use it, the competence of its consultants and its interface with leadership teams. MDC is a promising, systems-based program focused on building moral community by helping HCPs and other relevant teams encountering MoD to identify strategies to resolve recurring problems that create barriers to safe patient care and effective practice.

## Electronic supplementary material

Below is the link to the electronic supplementary material.

Supplementary Material 1

Supplementary Material 2
