# Supplementary material for: Moral Distress Consultation Services: Insights from Consultants
Source: HEC Forum. 2024 Aug 3;37(2):217–33. doi: 10.1007/s10730-024-09535-4 (PMC12014786; doi:10.1007/s10730-024-09535-4)
Supplement: Supplementary file 1 — Supplementary Material 1 [file 10730_2024_9535_MOESM1_ESM.docx]

Moral Distress Consultation Services: insights from consultants

HEC Forum

Vanessa Amos, MSN, RN, CNL,^1*^ Phyllis Whitehead, PhD, APRN, ACHPN, PMGT-BC, FNAP, FCNS, FAAN^2^, and Beth Epstein, PhD, RN, HEC-C, FAAN^3^

^1^ University of Virginia, (School of Nursing)

Charlottesville (VA), United States

ORCID ID: 0000-0001-7906-1663

^2^ Carilion Roanoke Memorial Hospital, (Palliative Medicine/Pain Management)

Roanoke (VA), United States

ORCID ID: 0000-0001-9530-1102

^3^ University of Virginia, (School of Nursing)

Charlottesville (VA), United States

ORCID ID: 0000-0001-7299-3214

*Corresponding Author Email: [vka7q@virginia.edu](mailto:vka7q@virginia.edu)

**Supplemental Table 1**

*Semi-Structured Interview Guide*

| Interview Questions |
| --- |
| 1. Can you walk me through how you know about the MDC service? |
| 1. Have you, yourself, had training in ethics or moral distress? If so, can you elaborate on it? |
| 1. How would you describe the purpose of the MDC service? |
| 1. Should there be contact with participants after a consult is held? |
| 1. In your opinion, are there drawbacks to having something like the MDC service, generally? Do you feel they can be well-implemented in bedside practice? In organizational practice? |
| 1. Do you feel consults will lead to change? Why or why not? |
| 1. Do you feel the organization supports interventions like the MDC service? How do they? Or how do they not? |
| 1. How do you feel the consult service could be made more visible to healthcare providers? To organizations? |
| 1. Can you describe what a consult would look like for it be “successful” to those involved in it? What about from an organizational perspective? |
| 1. What would you say needs to be in place for the MDC service to be sustainable? |
| 1. Is there anything else, related to the MDC service, MoD, or anything you’d like to elaborate on or discuss? |

*Note*. While the above questions served as a rough outline for all interviews, flexibility was permitted to change the conversation and/or interview focus based on an individual participant’s direction, request, or thought process.
