# Supplementary material for: Moral Distress Consultation Services: Insights from Consultants
Source: HEC Forum. 2024 Aug 3;37(2):217–33. doi: 10.1007/s10730-024-09535-4 (PMC12014786; doi:10.1007/s10730-024-09535-4)
Supplement: Supplementary file 2 — Supplementary Material 2 [file 10730_2024_9535_MOESM2_ESM.docx]

Moral Distress Consultation Services: insights from consultants

HEC Forum

Vanessa Amos, MSN, RN, CNL,^1*^ Phyllis Whitehead, PhD, APRN, ACHPN, PMGT-BC, FNAP, FCNS, FAAN^2^, and Beth Epstein, PhD, RN, HEC-C, FAAN^3^

^1^ University of Virginia, (School of Nursing)

Charlottesville (VA), United States

ORCID ID: 0000-0001-7906-1663

^2^ Carilion Roanoke Memorial Hospital, (Palliative Medicine/Pain Management)

Roanoke (VA), United States

ORCID ID: 0000-0001-9530-1102

^3^ University of Virginia, (School of Nursing)

Charlottesville (VA), United States

ORCID ID: 0000-0001-7299-3214

*Corresponding Author Email: [vka7q@virginia.edu](mailto:vka7q@virginia.edu)

**Supplemental Table 2**

*Candidate Themes and Exemplars*

| Candidate Theme | Exemplar(s) |
| --- | --- |
| **Training and introduction to the MDCS** | **Consultant 2080:** “Then there was a period where I would be the first call person and I would get the call and whatever came through I would have a backup, [someone] who is more experienced ethicist. And I would run the case by them and get advice from them. And then, over time, developed independence with the more simple consults.”  **Consultant 2556:** “And so, I started sitting in on some moral distress consults and got my training. I'd say in the trenches.”  **Consultant 3283:** “Well, I did a lot of ethics classes. And then actually, I can't remember the specific name of the conference, but [MDCS member] actually made me aware and let me work with you guys at [other institution]. I think they host like an ethics conference over a three-day weekend. That was pretty fantastic.” |
| **Defining purpose** | **Consultant 1558:** “I think it's a way to bypass local barriers and raise your hand really, really high and say, "This is an issue. I've tried to work within my little local realm and I need reinforcements." And this is a formal way to do that. To escalate things, bypassing the fear of retribution and the immediate power struggles.”  **Consultant 3494:** “[It’s a space for] having people open up and share moral distress and their feelings, whatever the particular situation is, and having folks who have not heard that before. So, it's an opportunity for folks to hear things that they didn't know about their co-workers, about their moral distress and how they felt. You know, it's not something that everybody shares.”  **Consultant 1017:** “And to feel listened to and heard by a neutral, or maybe not neutral, but someone who's not directly involved in the care team that they don't work with every day.” |
| **Interface with leadership** | **Consultant 1324:** “They [leadership] shouldn't be wary of it. They should be celebrating it. That it can help them have a stronger institution and help them be a better leader. So, education is a big thing.”  **Consultant 1032: “**And I think they [MDC participants] would feel most supported if they knew that they were heard and that their higher ups are going to make some change if that's needed.”  **Consultant 2556:** “I do think, having that respect of we [leadership] value the time that you're putting in. Because we have to have this.” |
| **Defining success** | **Consultant 3636:** “Because I think that it's hard a hard job for the facilitators to make people engaged, right? [For example] someone is really ragging on somebody else within the consult or there’s tension within the room and to some degree, that's still okay, we just need to work through it. So, I think the engagement is probably the number one thing and people really being interested.”  **Consultant 2080:** “It does seem like some of these things [subjects of MDC] are just hard circumstances that clinicians need to hear that they're not crazy. You know, like, diagnosing that, like, "No, this is a real problem. This isn't okay." And sometimes that almost seems like enough for people. Like [they'll say],"okay, alright, then I'll just deal with it the way I deal with it." |
| **Visibility and sustainability** | **Consultant 2556:** “I think for me, too, I try to be a walking advertisement. I'll say, "You know, an idea is looking at having a moral distress consult, let me tell you what that would look like." Does everybody call? Probably not.”  **Consultant 1558:** “I don't think it's utilized to its full potential. […] I know that there is work that is ongoing to try to help units that have been frequent fliers. To a certain degree, I think, in a perfect world, it [the MDC service] would eventually maybe work itself into not non-existence, but it would work itself into being more of a background player.”  **Consultant 1778:** “You've got to have tiers, right? To have the top person, you have to have the middle person that you're training to take over for the top person and then you have to have everybody to fill in the middle. You just got to build that system in place.” |
